# Prospective evaluation of voice outcome during the first two years in male patients treated by radiotherapy or laser surgery for T1a glottic carcinoma

**DOI:** 10.1007/s00405-012-1947-1

**Published:** 2012-02-05

**Authors:** Christine D. L. van Gogh, Irma M. Verdonck-de Leeuw, Jeanne Wedler-Peeters, Johannes A. Langendijk, Hans F. Mahieu

**Affiliations:** 1 Department of Otorhinolaryngology, Head and Neck Surgery, VU University Medical Center, P.O. Box 7057, 1007 MB Amsterdam, The Netherlands; 2 Department of Otorhinolaryngology, Maasland Hospital, Sittard, The Netherlands; 3 Department of Radiation Oncology, University of Groningen Medical Center, University of Groningen, Groningen, The Netherlands; 4 Voice Clinic, Department of Otorhinolaryngology, Meander Medical Centre, Amersfoort, The Netherlands

**Keywords:** Voice outcome, T1a glottic carcinoma, Radiotherapy, Laser surgery, Prospective results

## Abstract

In this prospective cohort study, we assessed voice outcome in patients before and up to 2 years after treatment for early glottic cancer either by radiotherapy or by laser surgery; 106 male patients, treated for T1aN0M0 glottic cancer either by endoscopic laser surgery (*n* = 67) or by radiotherapy (*n* = 39), participated in the study. Patients’ voices were recorded and analysed pre-treatment and 3, 6, 12 and 24 months post-treatment at their routine visit at the outpatient clinic. Average fundamental frequency (F0), percent jitter, percent shimmer and normalized noise energy (NNE) were determined. After 2 years, local control rate was 95% in the radiotherapy group and 97% in the laser surgery group. Larynx preservation rate was 95% after radiotherapy and 100% after laser surgery. Voice outcome recovers more quickly in patients treated with laser surgery in comparison to radiotherapy: 3 months after laser surgery there is no longer a difference with regard to normal voices except for the fundamental frequency, which remains higher pitched, even in the longer term. For patients treated with radiotherapy it takes longer for jitter, shimmer and NNE to become normal, where jitter remains significantly different from normal voices even after 2 years. According to these results, we believe that laser surgery is the first treatment of choice in the treatment of selected cases of T1a glottic carcinomas with good functional and oncological results.

## Introduction

Until about a decade ago, radiotherapy was the first choice of treatment for patients with early glottic cancer. Currently, it has been widely accepted that for these early stages endoscopic laser surgery can be a safe and valid alternative for radiotherapy. Cure rates are the major criterion in determining the treatment of choice. Since both treatment modalities provide good local control of approximately 90%, other criteria become important in determining the first treatment of choice [1–6].

One of these other criteria is the consideration that radiotherapy can be delivered only once at the same target area, while laser surgery can be performed repeatedly. Furthermore, radiotherapy takes a much longer period of treatment and recovery as compared to laser surgery. Therefore, in shared decision making in clinical practice, patients often prefer laser surgery. Another argument against radiotherapy is that laser surgery is much more cost-effective than radiotherapy [7–9].

Another important outcome measure is voice quality. Several cross-sectional studies have shown that voice outcome seems similar after both treatment modalities [10–17]. However, in most of these studies, information on tumour size, time of follow-up, and type of voice analyses is lacking. Moreover, prospective studies on voice outcome comparing both treatment modalities for comparable T1a lesions are scarce.

Therefore, the main purpose of this prospective cohort study was to assess voice outcome in patients before and up to 2 years after treatment for early glottic cancer either by radiotherapy or laser surgery. This study was approved by the local medical ethics committee.

## Patients and methods

### Patients

During a period of 9 years, 106 male patients were treated for T1aN0M0 (T1a: tumour limited to one vocal fold with normal mobility; N0: no regional lymph node metastasis; M0: no distant metastasis, according to the UICC staging system) glottic cancer. Staging was based on direct laryngoscopy and was proven by biopsy. Sixty-seven patients were treated by endoscopic laser surgery (mean age 66 years; range 34–87 years) and 39 by radiotherapy (mean age 65 years; range 44–85 years).

Twenty-one age-matched (mean age 64 years; range 50–81 years) males (spouses of patients visiting the outpatient clinic) without voice problems were used as controls.

#### Endoscopic laser surgery

Patients treated with endoscopic laser surgery were selected by means of videolaryngostroboscopic evaluation using the presence of mucosal undulation as an indication for superficial tumour spread only. A Sharplan CO_2_-laser (with ACU-spot micromanipulator; Sharplan Laser Industries, Tel Aviv, Israel) in a superpulse mode was used for a chordectomy Type II (according to the European Laryngological Society (ELS) classification [18]), involving resection of the epithelium, Reinke’s space and typically continuing the resection just into the deeper parts of the lamina propria. Because of this slight extension into the deeper parts of the lamina propria, this resection does not qualify as a type I resection, which is limited to Reinke’s space, the superficial part of the lamina propria.

#### Radiotherapy

Patients not selected for laser surgery were locally irradiated with the Varian CLINAC 2300, a linear 6 MV accelerator (Varia Medical Systems Inc., Palo Alto, CA, USA). The total radiation was 57.5–60.0 Gy (2.5 Gy per fraction, five times a week). All patients were treated with two opposing lateral fields, generally, with a standard field-size of 6 × 6 cm, using 6 MV photons.

### Methods

Patients’ voices were recorded and analysed pre-treatment and 3, 6, 12 and 24 months post-treatment at their routine visit at the outpatient clinic. Only patients were included in the present study of whom voice assessments of at least three of the assessment periods were completed and who had at least one voice assessment at 12 or 24 months. Patients who were treated for recurrence or suspicion of recurrence of the tumour during the follow-up period were excluded from the study.

### Acoustic voice analyses

Digital recordings of a sustained vowel /a/ at comfortable loudness and pitch were performed using Dr. Speech, developed by Tiger Electronics (Seattle, WA). A mouth-to-microphone distance of approximately 30 cm was held constant throughout all samples. Acoustic signal typing according to Behrman revealed that all recordings were suitable for further acoustic analyses [19]. Average fundamental frequency (F0), percent jitter, percent shimmer and normalized noise energy (NNE) were determined. The percentage of jitter represents the relative period-to-period variability. The percentage of shimmer represents the relative variability of the peak-to-peak amplitude. The normalized noise energy is the degree of noise produced by turbulent air escaping through the glottis during vocal emission.

### Statistical analyses

Independent *t* tests were used to compare the patient data versus the controls for all five assessment periods. Independent *t* test were also used in the comparison of voice results between the two different therapy groups. To investigate the longitudinal results for both treatment groups independently, paired *t* tests were used between the voice data of consecutive assessment periods.

## Results

### Patients

In total, 106 patients participated in the study. During the follow-up period, 10 patients underwent a complementary biopsy for suspicion of recurrence of the tumour and were excluded from further voice analyses. Three of them had been primary treated by radiotherapy, including two who had to be laryngectomised because of recurrence of tumour. The other patient who had no recurrence but merely moderate dysplasia was treated by laser surgery. Of the other seven patients, primary treated by laser surgery, two had tumour recurrence. One underwent radiotherapy, and the other one, laser surgery once more. The other five patients, primarily treated with laser surgery, suffered from light to moderate dysplasia and were treated once more by laser surgery (Table 1). None of the patients succumbed to their disease during the follow-up period. Another five patients were excluded for further analyses because they failed to complete the required number of at least three voice assessment moments even though they were not lost to oncological follow-up.

**Table 1:** Treatment outcome after 2 years

	Radiotherapy (*n* = 39)	Laser surgery (*n* = 67)	Total (*n* = 106)
Recurrence	2 (5%)	2 (3%)	4 (4%)
Larynx preservation	37 (95%)	67 (100%)	104 (98%)

Of the remaining 91 patients, 55 patients had been treated by endoscopic laser surgery (mean age 66 years; range 34–87 years) and 36 had been treated by radiotherapy (mean age 66 years; range 44–85 years). Median time of follow-up was comparable for patients treated with radiotherapy or laser surgery (Table 2).

**Table 2:** Median time of follow-up voice assessments in months after treatment

	3rd month assessment	6th month assessment	12th month assessment	24th month assessment
Radiotherapy				
Median	3.3	7.1	12.4	24.7
Laser surgery				
Median	3.6	6.8	12.4	24.5

### Voice outcome

**Fig. 1:**
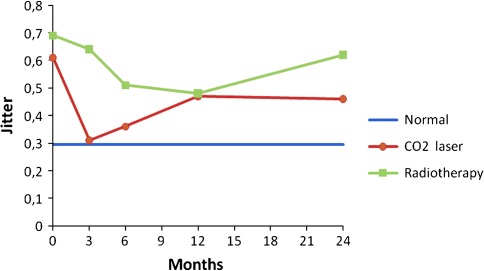
Prospective results of jitter for patients with T1a glottic carcinoma treated with either laser surgery or radiotherapy, compared with normal controls

**Fig. 2:**
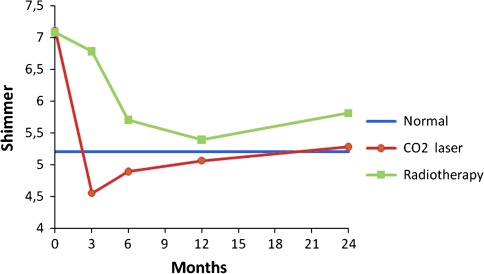
Prospective results of shimmer for patients with T1a glottic carcinoma treated with either laser surgery or radiotherapy, compared with normal controls

**Fig. 3:**
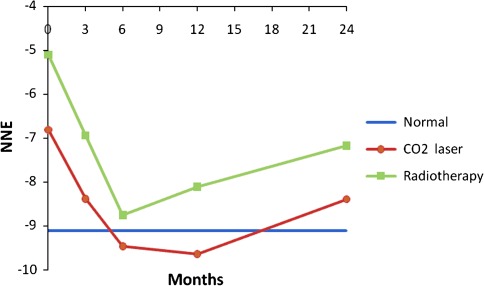
Prospective results of normalized noise energy for patients with T1a glottic carcinoma treated with either laser surgery or radiotherapy, compared with normal controls

**Fig. 4:**
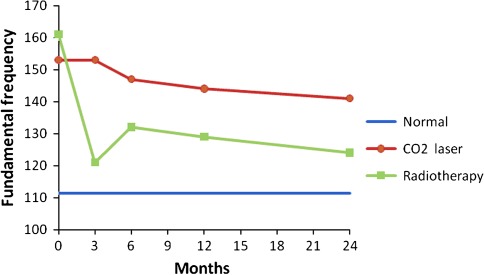
Prospective results of fundamental frequency for patients with T1a glottic carcinoma treated with either laser surgery or radiotherapy, compared with normal controls

Prospective voice outcomes are shown in Figs. 1, 2, 3, 4 of patients treated with radiotherapy (green lines) or laser surgery (red lines). Mean values of acoustic voice analyses of controls were jitter 0.30 (SD = 0.18), shimmer 5.20 (SD = 1.69), NNE −9.10 (SD = 3.21), and F0 111 Hz (SD = 24) and are represented by a blue line in Figs. 1–4.

In patients 3 months after radiotherapy, NNE was significantly better and the fundamental frequency was significantly lower compared to pre-treatment (*t* = 2.5, *p* = 0.021 and *t* = 4.2, *p* = 0.000 respectively). No significant voice changes occurred in the longer term at 6th, 12th and 24th month assessment.

In patients 3 months after laser surgery, jitter and shimmer were significantly better compared to pre-treatment (*t* = 3.2, *p* = 0.003 and *t* = 3.1, *p* = 0.004 respectively). No significant voice changes occurred in the longer term at 6th, 12th and 24th month assessment. Figures 1 to 4 represent the prospective results for jitter, shimmer, NNE and fundamental frequency.

### Patients versus controls

Before radiotherapy, patients scored significantly worse compared to controls regarding jitter (*t* = −3.1, *p* = 0.001), shimmer (*t* = −3.1, *p* = 0.003), and NNE (*t* = −4.3, *p* = 0.000) and fundamental frequency was significantly higher (*t* = −6.4, *p* = 0.000). Three months after radiotherapy, patients scored significantly worse regarding jitter, shimmer, and NNE (*t* = −3.0, *p* = 0.006; *t* = −2.1, *p* = 0.041 and *t* = −2.1, *p* = 0.042 respectively). Six and 12 months after treatment, patients scored significantly worse regarding jitter (*t* = −2.0, *p* = 0.050 and *t* = −2.4, *p* = 0.022 respectively) and fundamental frequency (*t* = −2.5, *p* = 0.016 and *t* = −2.4, *p* = 0.022 respectively) remained significantly higher compared to controls. Twenty-four months post treatment, jitter remained significantly worse in patients compared to controls (*t* = −2.8, *p* = 0.007).

Patients before laser surgery scored significantly worse compared to controls regarding jitter (*t* = −3.1, *p* = 0.003), shimmer (*t* = −2.5, *p* = 0.015), and NNE (*t* = −2.4, *p* = 0.21) and fundamental frequency was significantly higher (*t* = −6.0, *p* = 0.000). At 3, 6, 12 and 24 months after treatment, the fundamental frequency remained significantly higher in comparison with controls (*t* = −5.2, *p* = 0.000; *t* = −5.4, *p* = 0.000; *t* = −4.9, *p* = 0.000 and *t* = −4.2, *p* = 0.000 respectively); at these time points, all other voice outcome parameters were not significantly different between patients and controls. See also Table 3 and Figs. 1–4.

**Table 3:** Differences between treatment modalities regarding voice outcome

	Pre-treatment assessment		3rd month assessment		6th month assessment		12th month assessment		24th month assessment	
	RT	Laser	RT	Laser	RT	Laser	RT	Laser	RT	Laser
Jitter	0.69 (68)	0.61 (0.65)	**0.64** (0.55)	**0.31** (.22)	0.51 (0.54)	0.36 (0.30)	0.48 (0.41)	0.47 (0.75)	0.62 (0.62)	0.46 (0.49)
Shimmer	7.08 (2.73)	7.11 (4.76)	**6.78** (3.26)	**4.55** (1.98)	5.70 (2.54)	4.89 (2.75)	5.39 (2.66)	5.06 (4.46)	5.81 (3.75)	5.28 (3.19)
NNE	−5.10 (3.38)	−6.81 (4.77)	−6.94 (3.79)	−8.38 (3.90)	−8.57 (3.92)	−9.46 (4.43)	−8.11 (4.45)	−9.64 (5.09)	−7.17 (4.00)	−8.39 (4.23)
F0	161 (32)	153 (33)	**121** (29)	**153** (40)	132 (37)	147 (28)	**129** (32)	**144** (31)	**124** (29)	**141** (33)

Mean and (standard deviation); significant different outcomes are printed bold

### Radiotherapy versus laser surgery

Before treatment there was no significant difference for all four voice outcome parameters between patients treated with radiotherapy or laser surgery (Table 3).

Three months after treatment there was a significant difference between the two treatment modalities with better scores for patients treated with laser surgery regarding jitter and shimmer (*t* = −2.9, *p* = 0.007 and *t* = −3.1, *p* = 0.004 respectively) and higher fundamental frequency for patients treated with laser surgery (*t* = 3.8, *p* = 0.000). At 6, 12 and 24 months there were no significant differences any longer between the two treatment modalities except for the fundamental frequency. Voices of patients treated with laser surgery were significantly higher pitched compared to patients treated by radiotherapy at 12 and 24 months after treatment (*t* = 2.3, *p* = 0.027 and *t* = 2.4, *p* = 0.018 respectively) (Table 3).

## Discussion

In this study, four out of the 106 patients developed a recurrence, resulting in an overall local control of 96%. Overall larynx preservation rate was 98%. When comparing both treatment modalities local control rate after 2 years was 95% in the radiotherapy group and 97% in the laser surgery group. Larynx preservation rate was 95% after radiotherapy and 100% after laser surgery. Although it must be kept in mind that there is some selection bias because of the deliberate selection of tumours treatable by laser surgery (which implies the more superficial and less extensive tumours), it can be concluded that laser surgery for T1a glottic carcinomas results in excellent treatment outcome. Comparable results were found by other studies also including only T1a glottic laryngeal carcinomas as a homogenous study group. For example Sjögren et al [2] reported 5-year local control rates of 75% for patients after radiotherapy respectively 89% after laser surgery of T1a glottic carcinomas. In their study group, larynx preservation was also 100% for the laser treated patients versus 83% for the patients who received radiotherapy. Schrijvers et al. [20] also published a better larynx preservation rate of 95% for patients treated by laser surgery versus 77% for patients treated by radiotherapy after a follow-up of at least 41 months for T1a glottic carcinomas.

This paper describes a study investigating voice outcome prospectively from baseline to 2 years after treatment of patients treated with radiotherapy or laser surgery for T1a glottic carcinoma. Earlier studies most often involved retrospective analysis comparing measurements in a wide range of time intervals. The present study shows that recovery of the voice is dependent upon the time interval since the treatment, and that both treatment modalities result in a different recovery time regarding voice outcome. It appears that voice outcome recovers more quickly in patients treated with laser surgery in comparison to radiotherapy: 3 months after laser surgery there is no longer a difference with regard to the normal voices except for the fundamental frequency, which remains higher pitched, even in the longer term. For patients treated with radiotherapy it takes longer for jitter, shimmer and NNE to become normal, where jitter remains significantly different from the normal voices even after 2 years.

This current study provides evidence that, except from the fundamental frequency, in the long-term follow up there is no lasting difference in voice outcome between radiotherapy and laser surgery. After laser surgery the voices remain significantly higher pitched than after radiotherapy. This is in accordance with several other studies where the fundamental frequency also tends to be higher after laser surgery [11–13, 17]. This may be explained by increased stiffness of the vocal cord due to scar tissue after laser surgery and by a combination of scar tissue and edema after radiotherapy. Even before treatment the fundamental frequency is higher in both treatment groups than in normal male controls (F0 = 111 Hz, as found in present study) which can be attributed to a combination of increased vocal fold stiffness as a result of the tumour in combination with compensatory hyperkinetic voicing. This finding of a higher mean fundamental frequencies in patients with early glottic cancer has been demonstrated in other studies as well with mean fundamental frequencies varying from 151 to 204 [21–23].

It seems logical to expect that following endoscopic laser surgery the voice quality outcome highly depends on the extend and depth of the resection. Roh et al [21] divided his patients with early glottic cancer in different groups depending on the extent of laser surgery. He found that larger tumours and tumours involving the anterior commissure had poor voice quality. In our study, we only included T1a mid vocal cord tumours and pre treatment there were no significant differences in the voices between both treatment groups. In the light of this, it may very well be that patients with more extensive tumours, requiring more extended laser resections, are not better off after laser surgery in comparison to radiotherapy from a voice outcome point of view. Therefore, multidimensional decision making also taking into account the experience of the surgeon and the radiation oncologist remains an important issue.

Based on this study and supported by others in literature we believe that laser surgery is the first treatment of choice in the treatment of selected cases of T1a glottic carcinomas with good functional and oncological results.
